# Therapy with un-engineered naïve rat umbilical cord matrix stem cells markedly inhibits growth of murine lung adenocarcinoma

**DOI:** 10.1186/1471-2407-10-590

**Published:** 2010-10-28

**Authors:** Dharmendra K Maurya, Chiyo Doi, Atsushi Kawabata, Marla M Pyle, Clay King, Zhihong Wu, Deryl Troyer, Masaaki Tamura

**Affiliations:** 1 Department of Anatomy & Physiology, Kansas State University, College of Veterinary Medicine, Manhattan, KS 66506, USA

## Abstract

**Background:**

Lung cancer remains the leading cause of cancer-related mortality despite continuous efforts to find effective treatments. Data from the American Cancer Society indicate that while the overall incidence of lung cancer is declining, it continues to rise in women. Stem cell-based therapy has been an emerging strategy to treat various diseases. The purpose of this paper is to determine the efficacy of an intrinsic anti-cancer effect of rat umbilical cord matrix stem cells (UCMSCs) on lung cancer.

**Methods:**

A mouse syngeneic lung carcinoma model was used to test the basic ability of UCMSCs to control the growth of lung cancer. Lung tumors were experimentally induced by tail vein administration of Lewis lung carcinoma (LLC) cells derived from the lung of C57BL/6 mouse. Rat UCMSCs were then administered intratracheally five days later or intravenously on days 5 and 7. The tumor burdens were determined by measuring lung weight three weeks after the treatment.

**Results:**

Co-culture of rat UCMSCs with LLC significantly attenuated the proliferation of LLC cells as monitored by MTT (3-(4,5-Dimethylthiazol-2-yl)-2,5-diphenyltetrazolium bromide), a tetrazole cell proliferation assay, thymidine uptake, and direct cell counts. *In vitro *colony assays with rat UCMSCs as feeder layers markedly reduced LLC colony size and number. Co-culture of rat UCMSCs with LLCs causes G0/G1 arrest of cancer cells. This is evident in the decrease of cyclin A and CDK2 expression. The *in vivo *studies showed that rat UCMSC treatment significantly decreased tumor weight and the total tumor mass. Histological study revealed that intratracheally or systemically administered rat UCMSCs homed to tumor areas and survived for at least 3 weeks without any evidence of differentiation or adverse effects.

**Conclusions:**

These results indicate that rat UCMSCs alone remarkably attenuate the growth of lung carcinoma cells *in vitro *and in a mouse syngeneic lung carcinoma graft model and could be used for targeted cytotherapy for lung cancer.

## Background

Despite rapid advances in diagnostic and operative techniques, lung cancer remains one of the most difficult human malignancies to treat. The American Cancer Society estimates that 214,440 persons in the United States developed lung cancer in 2009, with 159,390 deaths [1]. Lung cancer-dependent deaths constituted 30% (men) and 26% (women) of the estimated total cancer-related deaths in 2009 [1]. Data from the American Cancer Society indicate that while the overall incidence of lung cancer is declining, it continues to rise in women [1]. The possible treatments for lung cancer include surgical resection, chemotherapy, radiotherapy, and/or combination therapy. Recently, multiple new chemotherapeutic agents have been developed and some are in clinical trials [2,3]. Although some of these have produced promising results, their therapeutic spectrum is narrow.

Genetically engineered mesenchymal stem cells derived from the umbilical cord matrix have great potential for therapeutic application in various diseases including cancer. However, a major advantage would be realized if tumor-trafficking stem cells that have not been genetically modified exhibit an inherent anti-tumor effect, thus circumventing the necessity of the expression of exogenous genes by the cells. It is well known that stem cells have inherent tumoritropic migratory properties [4]. Signals that mediate this effect appear to be similar or identical to those that mediate recruitment of stromal or defensive cells in tumors [5-7]. There are also a number of reports showing that genetically engineered stem cells efficiently deliver therapeutic proteins to cancer and other sites of inflammation [4,6,8-12]. Stem cells we have isolated from the Wharton's jelly of umbilical cord, termed 'umbilical cord matrix stem cells' (UCMSCs), also exhibit inherent tumoritropic migratory properties [8].

UCMSCs may be more useful for cancer therapy than other adult stem cells, since they are easy to prepare in relatively large quantities from umbilical cords after delivery and pose no ethical issues. They are potentially quite applicable to human patients without a complete genetic match, since they are unlikely to induce an acute immune response [13,14]. The versatility and availability of umbilical cord stem cells makes them a potent resource for transplant therapies for various diseases, including cancer. When these cells are engineered to secrete a cytokine, interferon beta (IFN-β), and are administered intravenously, they can attenuate metastatic breast cancer in a SCID mouse model [8]. Recently we found that rat UCMSCs completely abolished the growth of Mat B III cancer cells *in vitro *and *in vivo *[15]. Furthermore, un-engineered human UCMSCs have been shown to attenuate human breast cancer xenografts in a SCID mouse model [16]. Accordingly, the primary objective of the present study was to explore the therapeutic potential of rat UCMSCs against lung cancer using an LLC tumor model in syngeneic immunocompetent mice. This study surprisingly indicated that, even in trans-species transplantation, rat UCMSCs have exhibited a profound anti-tumor effect on lung carcinoma without any significant rejection of transplanted rat UCMSCs.

## Methods

### Cell culture

Rat UCMSCs were prepared from E19 pregnant rats and isolated using the method described previously [15]. Cells were maintained in defined medium, containing a mixture of 56% low glucose Dulbecco's Modified Eagle Medium (DMEM, Invitrogen, CA); 37% MCDB 201 (Sigma; St. Louis, MO); 2% fetal bovine serum (FBS, Atlanta Biologicals Inc, GA); 1x insulin-transferrin-selenium-X (ITS-X, Invitrogen); 1x ALBUMax1 (Invitrogen); 1x penicillin/streptomycin (Pen/Strep, Invitrogen); 10 nM dexamethasone (Sigma); 100 μM ascorbic acid 2-phosphate (Sigma); 10 ng/ml epidermal growth factor (EGF, R&D systems, Minneapolis, MN); and 10 ng/ml platelet derived growth factor-BB (PDGF-BB, R&D systems). Cells were maintained at 37°C in a humidified atmosphere containing 5% CO_2_.

The LLC cell line was maintained in DMEM (Invitrogen) medium supplemented with 10% FBS and 1x Pen/Strep (Invitrogen). Cells were cultured at 37°C in a humidified atmosphere containing 5% CO_2_.

### Cell proliferation assay

The MTT assay was performed to study the effect of rat UCMSCs on LLC cell proliferation. In brief, different ratios of rat UCMSCs and LLCs (UCMSCs:LLCs = 1:10, 1:6, and 1:3) in DMEM with 10% FBS were seeded in 96 well plates; cells were allowed to grow for 72 hrs. MTT solution (20 μl of 5 mg/ml) was added 4 hrs before completing 72 hrs of incubation. Formazan crystals formed were dissolved by adding 100 μl solublization buffer (10% SDS containing 0.01N HCl) and incubated overnight at 37°C. The following day, color developed was measured at 550 nm and background absorbance was measured at 630 nm using the Molecular Devices Spectramax 190 plate reader (Global Medical Instrumentation, Inc. Ramsey, MN).

### [^3^H] thymidine uptake assay

To evaluate cell proliferation from a different angle, a [^3^H] thymidine uptake assay was carried out. In brief, rat UCMSCs (1 × 10^3 ^or 2 × 10^3^/well) were mixed with 6 × 10^3 ^LLC cells/well, directly plated in 24-well culture plates, and cultured in a CO_2 _incubator for 72 hrs. Radioactivity incorporated into the cells was analyzed using our published protocol[15].

### Transwell cell culture study

Direct cell counts via hemocytometer were performed to study the effect of rat UCMSCs on LLC cell growth in culture plates with cell culture inserts (BD Biosciences, San Jose, CA). In brief, LLC cells were seeded in normal growth medium at 1 × 10^5 ^cells/well in 6-well plates. After allowing the cancer cells to adhere for 1 hr, 1.67 × 10^4 ^and 3.33 × 10^4 ^rat UCMSCs were seeded on the cell culture inserts (3.0 μm pore size). The insert pore size of 3 μm is small enough to prevent cells migrating from the inserts to the culture dishes, since UCMSCs do not penetrate this size pores in the insert. After 72 hrs co-culture, cells grown in the bottom culture dish were collected by trypsinization and counted using a hemocytometer.

### Colony formation study

Various numbers of rat UCMSCs (8.33 × 10^2 ^and 1.67 × 10^3 ^cells/well) were grown in a 12 well tissue culture plate. One day after seeding, 0.75 ml 0.8% agar (Sea Plaque agar, Cambrex Bio Science Rockland, Inc. Rockland, ME) in DMEM with 10% FBS was poured into the dish (bottom layer). After the bottom agar layer solidified, LLC cells (5 × 10^3 ^cells) were suspended in 0.5 ml of DMEM containing 10% FBS and 0.5% agar and plated on top of the bottom agar layer. The cells were incubated at 37°C with 5% CO_2 _for growth of colonies. On day 16, colony growth was evaluated by an automated phase contrast microscope equipped with Micro Suite Analysis Suite (Olympus CKX41, ***Center Valley, PA***). Colonies with an area greater than 50000 μm^2 ^were counted using Micro Suite Analysis Suite software.

### Cell cycle analysis

To analyze the effect of rat UCMSC co-culture on LLC cells, cell cycle analysis was carried out using propidium iodide staining. In brief, rat UCMSCs and LLC cells were co-cultured in 6 well Transwell culture dishes as described in the Transwell cell culture study. At the end of incubation LLC cells in the bottom chamber were collected and analyzed for cell cycle using our standard protocol [16].

### Western blot analysis

Total cellular protein was prepared using lysis buffer (1% TritonX-100, 0.1% SDS, 0.25M sucrose, 1 mM EDTA, 30 mM Tris-HCl (pH 8.0)) supplemented with protease inhibitor cocktail (Boehringer Mannheim, Indianapolis, IN). Protein samples were separated by a 10% SDS-PAGE gel, electroblotted onto nitrocellulose membrane (GE Healthcare, Uppsala, Sweden) and blocked with 4% nonfat dry milk in 0.1% Tween20 in phosphate buffered saline (PBST) for 1 hr at room temperature. The membranes were washed and incubated with antibodies against cyclin A (1:100, Abcam, Cambridge, MA), cyclin E (1:100, Abcam), and CDK2 (1:100, Santa Cruz Biotechnology, Santa Cruz, CA) with 4% nonfat dry milk in PBST for 1 hr at room temperature and then with a horseradish peroxidase-conjugated anti-rabbit IgG secondary antibody (GE Healthcare). The protein expression signal was detected with Pierce ECL Western Blotting substrate (Pierce, Rockford, IL). GAPDH was used as the loading control of sample by reprobing with an anti-GAPDH antibody (1:4000, Santa Cruz Biotechnology).

### Animals

Wild-type female C57BL/6 mice were obtained from the Jackson Laboratory (Bar Harbor, ME). All mice were housed in an AAALAC-accredited clean facility and held for 10 days to acclimatize. All animal procedures received prior approval from the Kansas State University Institutional Animal Care and Use Committee (protocol # 2681) and were performed in adherence with all applicable international, federal, state, and local guidelines.

### Systemic transplantation of LLC in lung and UCMSC treatment

To study the effect of rat UCMSCs on the growth of lung cancer grafts, a syngeneic LLC tumor model was used. In brief, each mouse was injected via the tail vein with 1.5 × 10^6 ^LLC cells (for Experimental design I) or 2 × 10^6 ^cells (for Experimental design II) suspended in 200 μl of PBS. On day 5 all mice were randomized into two treatment groups: a PBS control group and a rat UCMSC treatment group. To evaluate the trafficking of transplanted UCMSCs, cells were labeled with 10 μg/ml of SP-DiI fluorescent dye (Molecular Probes, CA) for 16 hrs incubation in a 5% CO_2 _incubator. After removing excess dye by washing the cells with PBS, cells were incubated with dye-free medium for another 4 hrs. These cells were dispersed by trypsinization and mixed with unlabeled UCMSCs so that 20% of the UCMSCs were labeled with SP-DiI. Although a preliminary cell culture study indicated that SP-DiI does not alter viability of UCMSCs, only 20% of transplanted cells were labeled in order to minimize any potential adverse effects of the dye. Two experimental designs were used for UCMSC injections.

### Experimental design I

five days after LLC cell injection, these mice were injected intratracheally using a 27 gauge needle with either 35 μl PBS (n = 8) or 35 μl rat UCMSCs suspension (3.5 × 10^5 ^cells in PBS, n = 9). The cell suspension in the syringe was released at approximately 5 mm above the dividing point of the bronchi. Our preliminary evaluations have shown that this injection method evenly distributes injected cells into the right and left lobes of the lung. **Experimental design II**: On day 5 and day 7 after LLC injection, these mice were systemically injected using a 28 gauge needle with either 200 μl PBS (n = 8) or 200 μl UCMSCs suspension (1 × 10^6 ^cells in PBS, n = 8) through the tail vein (IV).

All mice were kept in their cages and their physical condition was monitored until sacrifice on day 21 after LLC transplantation. Lungs of mice were collected immediately after sacrifice without inflation treatment, fixed in 10% formalin in saline, and used for histochemical analysis.

### Histopathology

Lung tissue fixed in 10% formalin in saline was embedded in paraffin, serially sectioned at 4 μm, and stained with hematoxyline and eosin (H&E) for microscopic examination of morphology of tumors. Unstained serial paraffin sections were counter stained with DAPI for nuclei and observed under epifluorescence microscopy for tracking the rat UCMSCs.

### Detection of apoptosis in tumors

To determine apoptosis in the tumors, the DeadEndTM colorimetric TUNEL system (Promega, Madison, WI) was used according to the manufacturer's protocol with a slight modification. The sections were counterstained with methyl green. To determine the apoptotic index, 10 nodules were selected randomly by light microscopy and the area of TUNEL positive cells in each nodule was calculated using the NIH Image J analysis software. The index was assessed as the percentage of TUNEL-positive area in the tumor. The fold change was calculated by dividing TUNEL-positive area in rat UCMSC treated tumors by those in untreated tumors.

### Statistical analysis

All values are expressed as means ± SE. For all *in vitro *and *in vivo *experiments, statistical significance was assessed by Tukey-Kramer Pairwise Comparisons test. All experiments were conducted at least twice with multiple sample determinations. Actual number of experiments repeated and sample numbers/experiments are described in the figure legends. Since all experiments were very closely reproduced, raw values were used for the statistical analysis. Data from replicate experiments as well as replicates within experiments were combined with no for experiment. A value of P < 0.05 was considered significant.

## Results

### Rat UCMSCs inhibit anchorage-dependent and -independent growth of LLC cells

**Figure 1:**
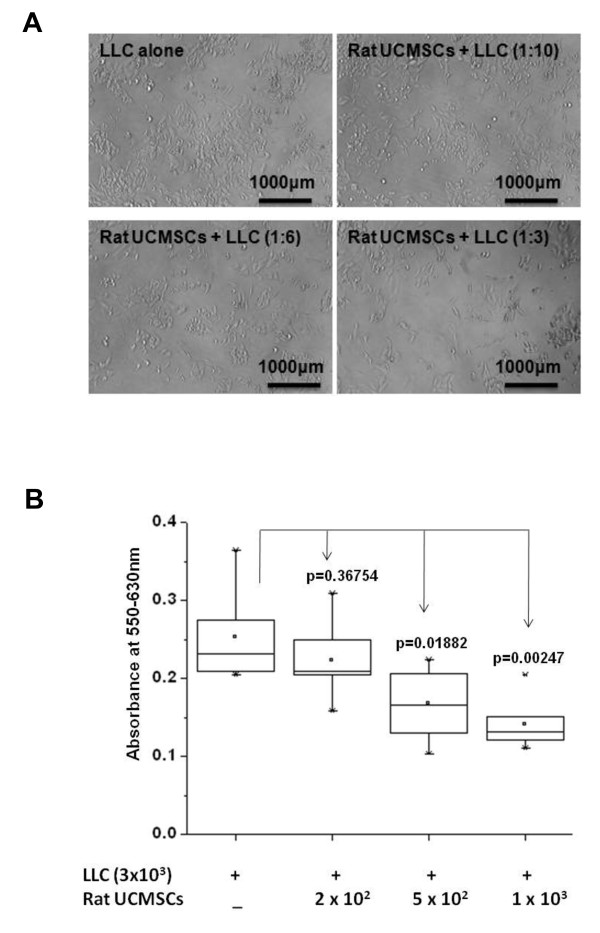
**Direct co-culture of a small number of rat UCMSCs significantly attenuated the growth of LLC cells**. Rat UCMSCs (300, 500 and 1000 cells/well) were co-cultured with 3 × 10^3 ^LLC (rat UCMSCs: LLC ratio, 1:10, 1:6 and 1:3). After three days of co-culture, the MTT assay was carried out. Panel A shows the morphology of LLC alone or LLC co-cultured with rat UCMSCs for 72 hrs. Scale bars in the pictures indicate 1000 μm. Panel B shows the summarized results from the MTT assay. The experiment was performed twice with 6 determinations at each point.

**Figure 2:**
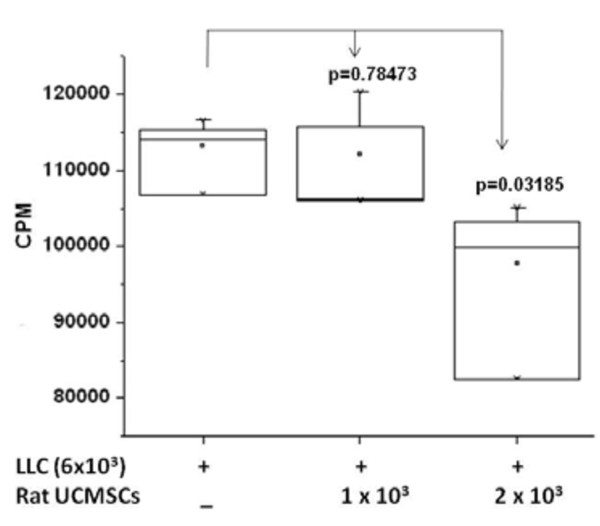
**[^3^H]-thymidine-uptake into LLC cells was significantly attenuated by co-culture with a small number of rat UCMSCs**. This experiment suggests that direct co-culture of a small number of rat UCMSCs significantly attenuates division of LLC cells. Rat UCMSCs (1 × 10^3 ^or 2 × 10^3^/well) were co-cultured with LLC cells (6 × 10^3^) in a 24-well culture plate. The [^3^H] thymidine-uptake was evaluated 72 hrs after the co-culture, as described in the Materials and Methods section. The experiment was performed twice with quadruplicate determinations.

**Figure 3:**
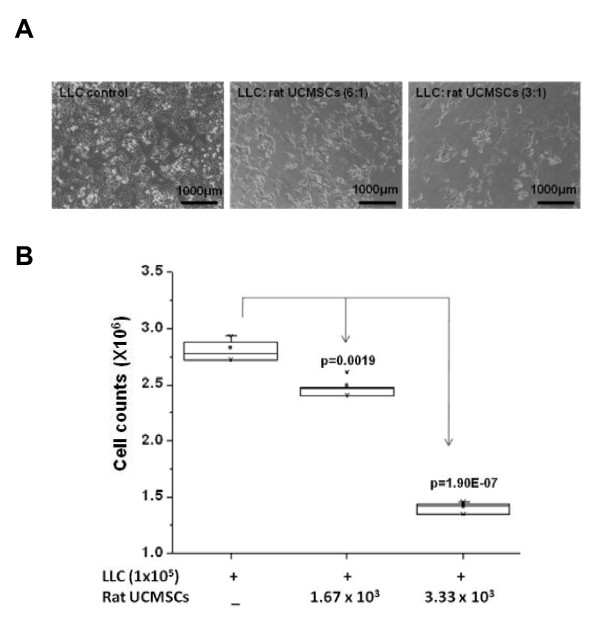
**Rat UCMSCs significantly attenuated the growth of LLC cells in a Transwell co-culture**. This experiment suggests that indirect co-culture of a small number of rat UCMSCs significantly attenuates the growth of LLC cells. Panel A shows the morphology of LLC alone or LLC indirectly co-cultured with rat UCMSCs for 72 hrs. Panel B shows the summarized results from direct cell counts using a hemocytometer. The experiment was performed twice with triplicate determinations.

The effect of un-engineered rat UCMSCs on anchorage-dependent growth of LLC cells was evaluated by both direct and indirect co-culture. As shown in Figure 1, direct co-culture of rat UCMSCs with LLC cells (ratio, 1:6 or 1:3) markedly decreased the total LLC cell number as measured by MTT assay. DNA synthesis as determined by thymidine uptake assay was in good agreement with the MTT assay result (Figure 2), although significant growth inhibition of LLC cells by rat UCMSCs was observed with a smaller proportion of UCMSCs in the MTT assay (UCMSC:LLC = 1:6) than in the thymidine uptake assay (UCMSC:LLC = 1:3). In addition to these studies in which both cell types directly contacted each other, an indirect co-culture study was carried out using a Transwell culture system in which rat UCMSCs were cultured in inserts and LLC cells were cultured in the bottom of the well. The effect of this indirect co-culture was assessed by counting the LLC cells after 72 hrs co-culture. The findings of this experiment (Figure 3) also corroborate with the results from the MTT and thymidine uptake assays. Un-engineered rat UCMSCs dose-dependently attenuated the total number of LLC cells. This Transwell experiment suggests that rat UCMSC-dependent cell growth attenuation may be mediated through a diffusible molecule or molecules produced by rat UCMSCs. Another Transwell cell culture in which LLC cells (1 × 10^5 ^cells) were cultured in the inserts and rat UCMSCs (1.7 × 10^3^) were cultured in the bottom of the wells revealed that LLC cells do not alter the growth of rat UCMSCs (data not shown).

**Figure 4:**
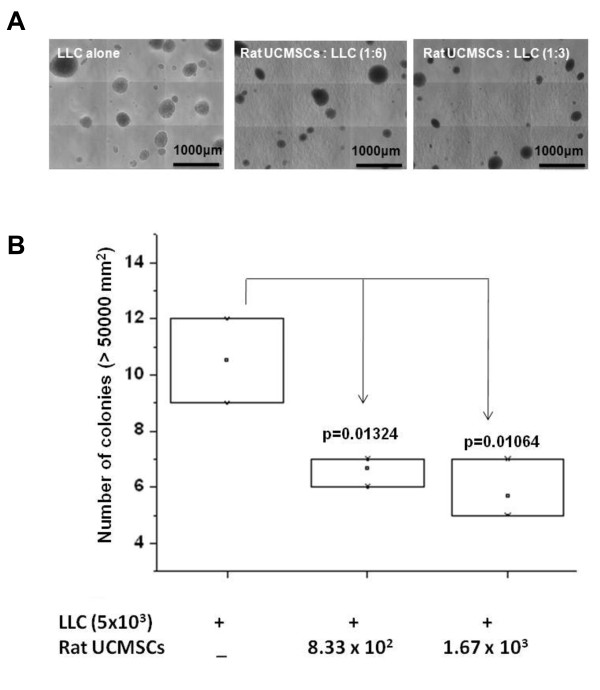
**Rat UCMSCs significantly attenuated colony growth of LLC cells**. Panel A shows the morphology of colonies of LLC alone or LLC co-cultured with rat UCMSCs in soft agar after 16 days. Panel B shows the summarized results from the colony assay. The experiment was performed twice with triplicate determinations.

Since anchorage-independent growth is a hallmark of tumorigenesis, a soft agar assay was carried out to evaluate the effect of rat UCMSCs on the growth of LLC cell colonies. The soft agar colony assay is an *in vitro *model to mimic *in vivo *xenograft models. The results showed that LLC cell colony size and number in soft agar were significantly attenuated when rat UCMSCs were cultured in the bottom of the culture dish (Figure 4). Since LLC cells were separated from rat UCMSCs by a solid agar layer (approximately 1 mm thickness), and the two cell types physically do not contact each other, the colony assay results also suggest that rat UCMSC-dependent growth attenuation was mediated through molecules produced by rat UCMSCs which diffused to LLC cells.

### Rat UCMSCs induced G0/G1 cell cycle arrest

**Figure 5:**
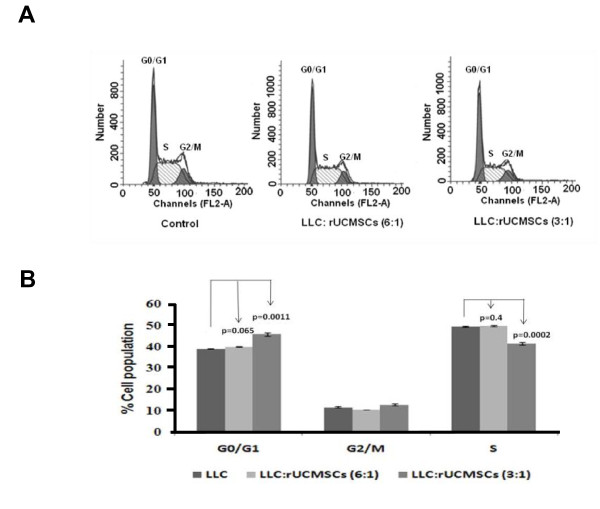
**Indirect co-culture of a small number of rat UCMSCs with LLC cells in a Transwell cell culture caused G0/G1 arrest in LLC cells**. In the co-culture study, 1.67 × 10^4 ^or 3.33 × 10^4 ^rat UCMSCs were placed in the culture plate inserts and co-cultured with 1 × 10^5 ^LLC cells in the bottom of the culture plates. After two to three days co-culture, LLC cells were collected from the bottom chamber by trypsinization and subjected to flow cytometry as described in Materials and Methods. Panel A represents the flow cytometry result and panel B represents the histogram pattern of different phases of the cell cycle. The experiment was performed twice with triplicate determinations.

**Figure 6:**
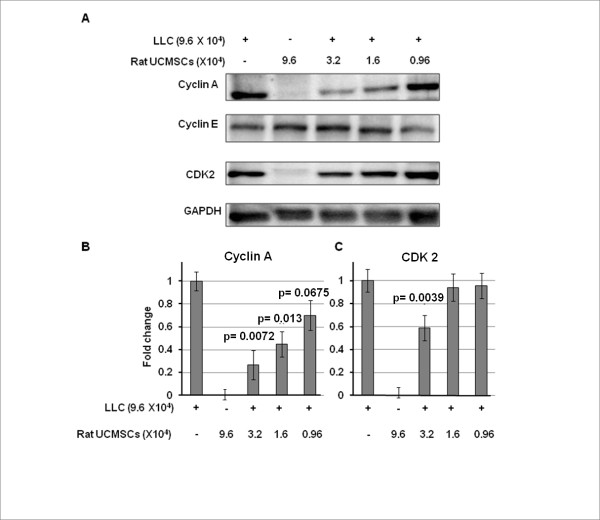
**Rat UCMSCs significantly attenuated protein expression of both cyclin A (Panels A and B) and CDK2 (Panels A and C), but not cyclin E (Panel A), in co-cultured LLC cells**. This experiment suggests that indirect co-culture of a small number of rat UCMSCs caused G0/G1 arrest in LLC cells. Samples were prepared as described in the Figure 5 legend and in Materials and Methods, and subjected to Western blot analysis. Sample preparation and Western blot analysis were performed three times with duplicate determinations. The pictures (Panel A) represent typical blotting results. P values were calculated as compared to the level of control.

The effect of rat UCMSCs on the cell cycle of LLC cells was evaluated by flow cytometry after the LLC cells were co-cultured with rat UCMSCs in Transwell culture dishes. The result shows that rat UCMSCs caused G0/G1 arrest of LLC cells, thus increasing the G0/G1 population and decreasing the S phase population of LLC cells (Figure 5A). As shown in Figure 5B, the G0/G1 phase cell populations (%) in LLC alone, LLC:UCMSCs (6:1), and LLC:UCMSCs (3:1) were 39.02 ± 0.06, 39.89 ± 0.11, and 45.84 ±0.81, respectively; S phase populations (%, same order as above) were 49.35 ± 0.24, 49.69 ± 0.25, and 41.33 ±0.63, respectively. Furthermore, cyclins and cyclin-dependent kinase were analyzed by Western blot analysis. As shown in Figure 6, co-culture with rat UCMSCs significantly attenuated protein expression levels of cyclin A and CDK2, but not cyclin E. These results also indicate cell cycle arrest in G0/G1 phase.

### Un-engineered rat UCMSCs significantly attenuate growth of LLC grafts in syngeneic mice

**Figure 7:**
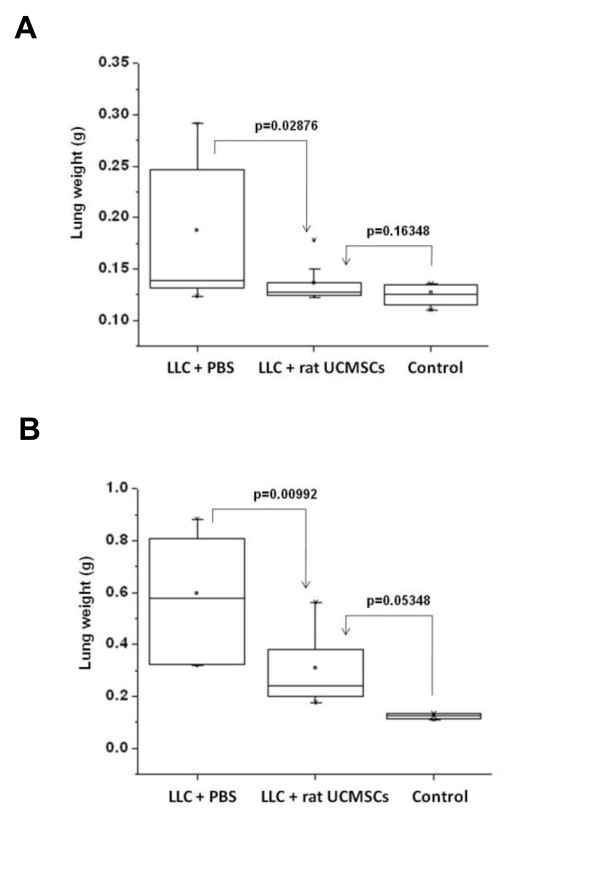
**Administration of rat UCMSCs significantly attenuated the growth of LLC tumors in a syngeneic mouse model**. Panel A shows that lung weight in mice transplanted with one million LLC cells declines almost to non-cancerous control levels after a single intratracheal administration of rat UCMSCs (0.35 million cells). Panel B shows that systemic transplantation of rat UCMSCs (one million cells twice) also significantly decreased the weight of the LLC-bearing lungs despite a marked increase of tumor burden due to a larger number of LLC cells transplanted (two million cells). In both experiments, eight mice per group were used except for the rat UCMSCs intratracheally administered group, which consisted of nine mice.

**Figure 8:**
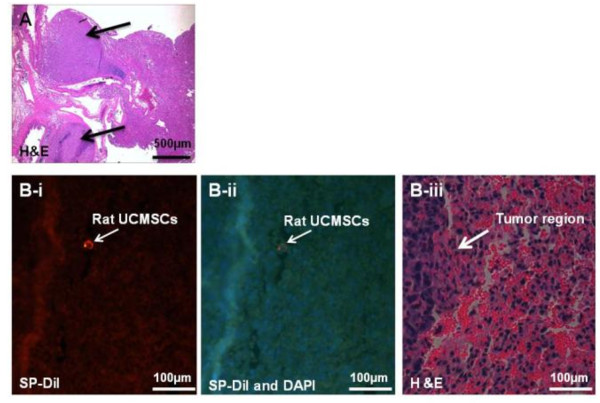
**SP-DiI labeled rat UCMSCs administered intratracheally were detected near the tumors**. This experiment suggests that intratracheally administered rat UCMSCs home at adjacent to the lung tumor area. Serial sections from paraffin-embedded lung were used for the detection of rat UCMSCs labeled with SP-DiI fluorescent dye either with SP-DiI fluorescence alone (B-i) or with both DAPI nuclear staining and SP-DiI fluorescence (B-ii). Tumor morphology was observed after H&E staining (A, PBS-treated tumors, arrows indicate tumors, and B-iii, rat UCMSC-treated tumor, arrow indicates tumor margin). Scale bars in Panel A and B indicate 500 μm and 100 μm, respectively.

We tested the growth attenuation ability of un-engineered naïve rat UCMSCs on the lung LLC tumors by local (intratracheal) and systemic (IV) administration. Results indicate that a single intratracheal administration of rat UCMSCs 5 days after LLC transplantation almost completely inhibited tumor growth as measured by tumor weight (Figure 7A). The IV administration of rat UCMSCs also significantly attenuated tumor growth despite the fact that tumor growth was more prominent due to a larger number of the LLC cells transplanted (2 million cells/mouse) (Figure 7B). Histological analysis revealed that lungs of PBS injected mice were occupied by large amounts of cancer tissue, whereas rat UCMSC-treated groups contained almost negligible (intratracheal injection group) or very small amounts of tumor mass (systemic injection group). Fluorescence microscopic observation of serial sections used for H&E staining shows the engraftment of rat UCMSCs in close proximity to or within tumor tissues of tumor bearing mice that received SP-DiI labeled rat UCMSCs either intratracheally or systemically (Figure 8). However, SP-DiI labeled rat UCMSCs were not detected in normal areas of the lung.

### Rat UCMSC treatment markedly increased apoptosis in tumor tissues

**Figure 9:**
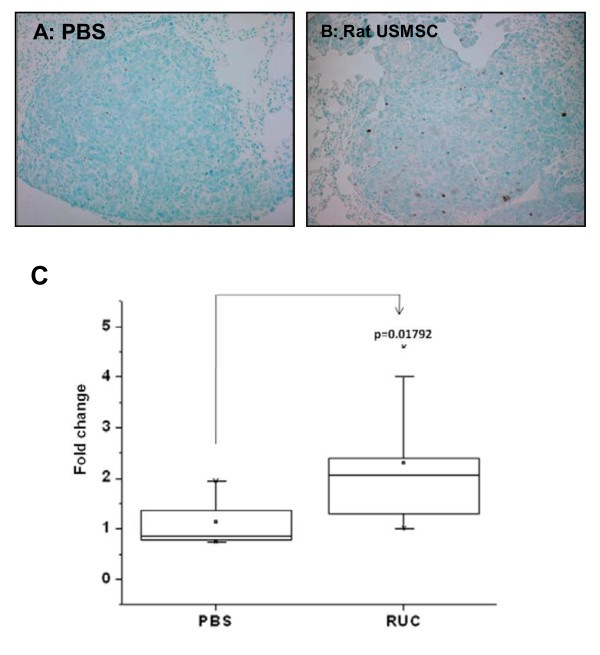
**Analysis of apoptosis in LLC tumors in syngeneic mouse lungs treated with either PBS (A) or rat UCMSCs (B) indicates that rat UCMSCs increase apoptotic cell death**. Lungs were collected, and apoptosis in tumors was analyzed by evaluating the area of TUNEL positive cells (panels A and B) as described in Materials and Methods. The average TUNEL positive area(C) were determined by analyzing ten tumor areas in each treatment group and expressed as fold change compared to PBS-treated controls.

To evaluate the effect of the rat UCMSCs on the apoptotic activities of tumor cells *in vivo*, the percentage of TUNEL-positive cell area in the tumors was determined. The percentage of TUNEL-positive cell area was two times higher in rat UCMSC treated tumors than in PBS treated control tumors (Figure 9). These results indicated that treatment with un-engineered rat UCMSCs increases apoptosis significantly.

## Discussion

The present study shows for the first time that naïve rat UCMSCs have the potential to attenuate rapidly growing murine lung carcinoma cells in a syngeneic mouse model. This effect was observed in most tumor-bearing mice transplanted either locally or systemically with rat UCMSCs. In addition, we describe here the following important new findings: 1. in co-culture, a relatively small number of rat UCMSCs significantly attenuated proliferation of LLC cells; 2. *in vitro *three dimensional LLC colony formation is markedly attenuated when rat UCMSCs are present as a monolayer in the bottom of the culture; 3) rat UCMSCs caused G0/G1 arrest in LLCs, and this may be a possible mechanism of growth attenuation; 4) either local or systemic rat UCMSC therapy significantly decreased the lung tumor burden; 5) rat UCMSCs administered intratracheally or IV 'home' into or near the lung tumors

Attenuation of tumor cell growth by rat UCMSCs appears to have both contact-independent and -dependent components. Interestingly, Khakoo *et al*. showed that bone marrow mesenchymal stem cells could only mediate their effect on Kaposi sarcoma by contact with the tumor cells *in vitro *[17]. Here we showed that in co-culture assays (Figures. 1 and 2); rat UCMSCs co-cultured with LLC cells significantly attenuate LLC cell proliferation. Since the number of rat UCMSCs was significantly smaller than LLCs (1:6 or 1:3), this rat UCMSC-dependent effect is not strictly contact-mediated; it is likely that some factor(s) independent of cell-to-cell contact is involved. In the Transwell co-culture study and soft agar colony assay, in which LLC cells were not in direct contact with rat UCMSCs (Figures. 3 and 4), the LLC growth inhibition by rat UCMSCs implies involvement of a diffusible factor or factors secreted by the stem cells. This implication is in good agreement with previous studies from our laboratory in which rat UCMSCs attenuated growth of rat mammary carcinoma cells, apparently through diffusible molecules [15]. This factor may be associated with the regulation of the cell cycle, since the present study clearly indicated that rat UCMSCs induced G0/G1 arrest in LLC cells when they were co-cultured in a Transwell culture system (Figure 5), in which the two cell types do not have direct contact. Furthermore, the anti-tumor effect shown here may also be associated with pro-apoptotic factors produced by naïve rat UCMSCs (Figures. 6 and 9). Based on the present study, cell cycle regulation- and/or apoptosis-associated genes may be good candidates. However, an involvement of other mechanisms, such as a stimulation of the host immune system, may also be a part of tumor growth attenuation *in vivo*.

The homing ability of stem cells has previously been exploited for drug delivery and targeted gene delivery [5,6,8-10,12,18]. The homing of stem cells to tumors and other areas of inflammation is well established [5-8,19]. The homing ability of stem cells appears to be mediated by chemokines secreted by the tumors or their associated stroma [20-22]. Potential chemokines could include growth factors such as platelet derived growth factor (PDGF) family members and epidermal growth factor (EGF) [23] in addition to classical chemo-attractants such as CXCR4 and its ligand, SDF-1. In the present study, SP-DiI-loaded rat UCMSCs were detected either within the tumors or adjacent to the tumors (Figure 8). Potential reasons why only a few dye loaded UCSMCs were detected in lung tumor sites follow: 1) lung tissue was fixed with 10% formalin, and formalin is known to have a fluorescence quenching effect; 2) only 20% of UCMSCs were labeled by SD-DiI; and 3) the chances of encountering a small number of UCMSCs in a 4 μm section are relatively low even if a large number of cells were localized in the entire tumor area. Thus, detection of even a small number of SP-DiI-loaded rat UCMSCs in or adjacent to the tumors may imply that rat UCMSCs were attracted to the chemokines produced by tumor tissues and homed to the tumor tissues.

The ability of naïve UCMSCs to eliminate lung adenocarcinomas is a distinct advantage, since any manipulation causing the cells to express an exogenous gene could alter them in some way that would potentially make them less safe as transplantable cells. However, since rat UCMSCs are not directly applicable to human therapy, key mechanism(s) by which rat UCMSCs exhibit their powerful anti-tumor effect should be identified first. This mechanism may be applied to human UCMSCs for future human application. Therefore, it will be worthwhile to identify which genes or gene products make rat UCMSCs so powerful for attenuation of lung cancer growth. If human UCMSCs are as potent as rat UCMSCs, human UCMSCs will potentially be utilized for human cancer therapy. If human UCMSCs are not as potent as rat UCMSCs, their cytotoxicity to cancer cells may be enhanced by manipulation of their gene expression based on the study of rat UCMSC-dependent cytotoxicity against lung cancer.

In this study we have used rat origin UCMSCs for a mouse cancer model and they have shown a very strong effect. Whether the rat UCMSC-dependent anti-tumorigenetic effect is partially due to the xenotransplantation is unclear. However, since rat UCMSCs were extremely potent in cell growth attenuation in simple *in vitro *cell culture studies where no immune cells or immunoglobulin were involved, it is likely that their *in vivo *effect is also independent of nonspecific immune surveillance induced by xenotransplantation. In support of this, histopathological analysis did not show lymphocyte infiltration in tumor tissues (data not shown). In addition, our previous finding that human UCMSCs are poorly immunogenic [14] also indirectly supports the above speculation.

Naïve UCMSCs have many potential advantages for cytotherapy. Among many tissue-originated multipotent stem cells, UCMSCs are very usable due to their abundance, low immunogenicity [14], lack of CD34 and CD45 expression, and simplicity of the methods for harvest and *in vitro *expansion [12,24,25]. These properties of UCMSCs encourage their development as therapeutic tools or agents because they can potentially be used for allogeneic transplantation.

## Conclusions

In *in vitro *studies, co-culture of rat UCMSCs significantly attenuated the proliferation of LLC cells as monitored by MTT assay, thymidine uptake, and direct cell counts. Rat UCMSCs also markedly reduced LLC colony size and number. Co-culture of rat UCMSCs caused G0/G1 arrest of LLC cells. This is evident in the decrease of cyclin A and CDK2 expression. The *in vivo *studies showed that rat UCMSC treatment significantly decreased tumor weight and the total tumor area. Histological study revealed that intratracheally or systemically administered rat UCMSCs homed to tumor areas and survived for at least 3 weeks without any evidence of differentiation or adverse effects. Thus the findings described here suggest that UCMSCs may represent a new therapeutic modality for the treatment of lung cancer and will have important implications for patients with lung cancer and other types of cancer.

## List of abbreviations

DAPI: 4',6-diamidino-2-phenylindole; EGF: epidermal growth factor; FBS: fetal bovine serum; H&E: hematoxyline and eosin; IFN-β: Interferon beta; ITS-X: insulin-transferrin-selenium-X; LLC: Lewis lung carcinoma; MTT: Methylthiazol Tetrazolium; PDGF: platelet derived growth factor; PDGF-BB: platelet derived growth factor-BB; SDF-1: stroma derived factor-1; UCMSCs: umbilical cord matrix stem cells; CDK2: Cyclin-dependent Kinase 2; GAPDH: Glyceraldehyde 3-phosphate dehydrogenase;

## Competing interests

The authors declare that they have no competing interests.

## Authors' contributions

DKM, CD, AK, CK, ZW, and MT were responsible for the study design, experimental work, data evaluation and analysis, and drafting the manuscript. MMP and DT were consulted extensively in the experimental design and interpretation of results, as well as in the preparation of the manuscript. MT was the research supervisor and participated in the study design, assessment of the results, and drafting the manuscript. All authors read and approved the manuscript.

## Pre-publication history

The pre-publication history for this paper can be accessed here:

http://www.biomedcentral.com/1471-2407/10/590/prepub
